# Case Report: Reversible acquired Chiari-like state and upper cervical syringomyelia caused by a giant posterior tentorial tumor confined to the supratentorial compartment

**DOI:** 10.3389/fsurg.2026.1881912

**Published:** 2026-07-15

**Authors:** Pablo Albiña-Palmarola, Ali Khanafer, Gottlieb Maier, Ketevan Mikeladze, Philipp von Gottberg, Oliver Ganslandt, Hans Henkes

**Affiliations:** 1 Neuroradiologische Klinik, Kopf- und Neurozentrum, Klinikum Stuttgart, Stuttgart, Germany; 2 Neurochirurgische Klinik, Klinikum Stuttgart, Stuttgart, Germany; 3 Medizinische Fakultät, Universität Duisburg-Essen, Essen, Germany

**Keywords:** acquired Chiari, case report, Chiari-like state, craniocervical hydrodynamics, solitary fibrous tumor, syringomyelia

## Abstract

Acquired tonsillar descent with syringomyelia secondary to intracranial space-occupying lesions is rare, and supratentorial causes are particularly uncommon. These cases are of particular pathophysiological interest because they suggest that tonsillar descent and syrinx formation may arise as downstream consequences of altered intracranial compliance, compartmental pressure transmission, and impaired craniocervical cerebrospinal fluid buffering, rather than from a primary hindbrain malformation. We report a previously healthy 39-year-old woman who presented with progressive headache, papilledema, and left homonymous hemianopia. Imaging demonstrated a giant posterior tentorial dural-based mass confined to the supratentorial compartment, with marked mass effect, inferior tentorial deformation, 7-mm caudal tonsillar descent below McRae's line, and a small C1 syrinx. The patient underwent preoperative embolization and near-total microsurgical resection, followed 6 weeks later by single-fraction LINAC-based stereotactic radiosurgery to a small residual remnant. Follow-up MRI showed reduction of the remnant, resolution of the midline shift, restoration of normal tonsillar morphology and position, and complete resolution of the syrinx, accompanied by substantial visual recovery. The observed reversal after treatment of the causative lesion supports a model in which the supratentorial mass secondarily disturbed craniocervical hydrodynamics and produced a reversible acquired Chiari-like state with upper cervical syringomyelia. This case highlights the need for careful cranial imaging before posterior fossa decompression is considered in adults with newly identified tonsillar descent and syringomyelia.

## 1 Introduction

Tonsillar ectopia with syringomyelia is usually discussed within the framework of Chiari I malformation. Yet the radiological phenotype of “Chiari I” is mechanistically heterogeneous, and secondary forms have long been recognized in association with cerebrospinal fluid (CSF) diversion, cranial disproportion, intracranial hypotension, hydrocephalus, and space-occupying lesions (1, 2). In such cases, the descent of the cerebellar tonsils is better understood as a downstream mechanical or hydrodynamic consequence than as a primary developmental hindbrain disorder (1, 3).

The literature on acquired Chiari-like state caused by intracranial lesions remains limited. In the largest systematic review, Wang et al. identified only 44 patients with acquired Chiari malformation and associated syringomyelia secondary to space-occupying lesions; 89% of the lesions were infratentorial, 82% of patients had syringomyelia, and improvement generally followed treatment of the underlying lesion rather than syrinx-directed surgery (2). Supratentorial examples are sparse and heterogeneous, but the published cases consistently reinforce the same principle: if the hindbrain and spinal cord findings are secondary, the primary pathology should be addressed first (4–7). The present case provides radiological evidence of a reversible acquired Chiari-like state with upper cervical syringomyelia caused by a supratentorial lesion, supporting the interpretation that these hindbrain and cervical cord findings represented a secondary craniocervical hydrodynamic disorder rather than a primary Chiari malformation, and providing an opportunity to re-examine the complex pathophysiological mechanisms implicated in this sequence.

## 2 Case description

A 39-year-old previously healthy woman presented to the emergency department with a 9-month history of persistent, progressively worsening headache associated with nausea and blurred vision. During the month preceding admission, she developed increasing difficulty seeing objects in her left visual field. Neurological examination demonstrated bilateral papilledema and a left homonymous hemianopia. The remainder of the neurological examination was unremarkable. Goldmann perimetry confirmed a complete left homonymous hemianopia. Emergency contrast-enhanced computed tomography (CT) demonstrated a large, avidly enhancing right temporoparietooccipital extra-axial lesion with marked mass effect and midline shift, without significant hydrocephalus or evidence of recent hemorrhage (Figure 1A). There was no adjacent hyperostosis, osseous erosion, or intratumoral calcification. The patient was therefore admitted for further evaluation and treatment.

**Figure 1:**
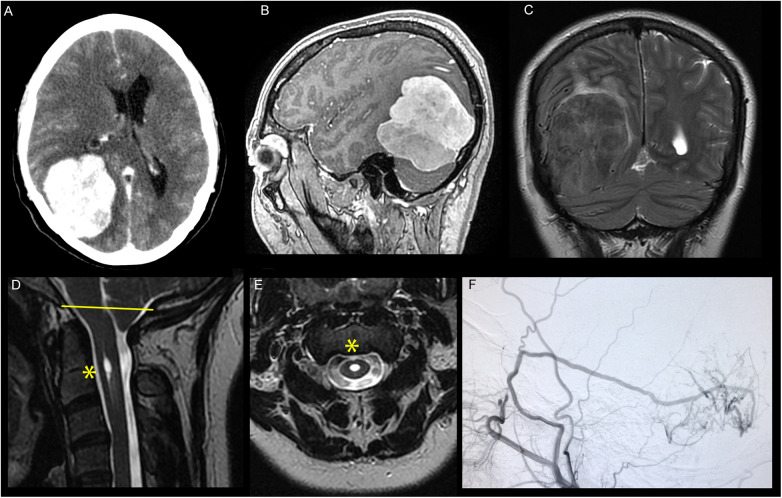
Preoperative imaging and angiography. **(A)** Axial contrast-enhanced CT scan demonstrating a large, avidly enhancing right temporoparietooccipital extra-axial mass with marked mass effect and midline shift, without hydrocephalus. **(B)** Sagittal post-contrast T1WI. **(C)** Coronal T2WI showing a posterior tentorial dural-based mass confined to the supratentorial compartment, displacing the tentorium inferiorly. Dural involvement is suspected, and the patency of the transverse sinus is uncertain. **(D)** Midline sagittal T2WI of the craniocervical junction demonstrating peg-like caudal impaction of the cerebellar tonsils, measuring 7 mm below McRae's line (yellow line), and a small syrinx at the C1 level (asterisk). **(E)** Axial T2WI at C1 confirming the small syrinx. **(F)** Right external carotid artery angiogram, arterial phase, demonstrating marked tumor blush and corkscrew feeding vessels.

Magnetic resonance imaging (MRI) confirmed a 4.9 × 7.1 × 7.0-cm well-defined, lobulated extra-axial mass arising from the superior surface of the posterior tentorium and confined to the supratentorial compartment. Radiologically, the lesion was isointense to gray matter on T1-weighted imaging (T1WI), heterogeneously hyperintense on T2-weighted imaging (T2WI), and showed intense enhancement after gadolinium administration, with a narrow dural tail (Figures 1B,C). Arterial spin labeling (ASL) perfusion suggested a hypervascular lesion, and mild perilesional edema was also present. Most importantly, there was severe regional mass effect, with a 7-mm midline shift and ipsilateral subfalcine and uncal herniation. Despite the supratentorial location of the lesion, the tentorium appeared slightly displaced inferiorly. In addition, sagittal MRI of the craniocervical junction demonstrated peg-like caudal descent of the cerebellar tonsils, extending 7 mm below McRae's line, together with a small syrinx centered at the C1 level (Figures 1D,E). No additional posterior fossa structural abnormality was identified. Digital subtraction angiography (DSA) confirmed a hypervascular tumor blush supplied by both dural and pial branches, including feeders from the right middle meningeal, occipital, and posterior auricular arteries, as well as a hypertrophic temporo-occipital branch of the middle cerebral artery and branches of the posterior cerebral artery (Figure 1F). No venous sinus thrombosis was identified. Thoracoabdominal imaging showed no evidence of additional extracranial lesions. The preoperative imaging appearance suggested a primary dural-based neoplasm, with meningioma and solitary fibrous tumor (SFT) as the main differential considerations. Given the clinical syndrome of intracranial hypertension and progressive retrochiasmal visual dysfunction, urgent treatment was undertaken.

Partial preoperative embolization was performed 1 day before surgery through the posterior branch of the middle meningeal artery using 100–300-*μ*m microspheres to reduce tumor vascularity and facilitate resection. No embolization-related complication occurred. The patient subsequently underwent a right temporoparietooccipital craniotomy for microsurgical tumor removal (Figure 2). Intraoperatively, the lesion was confirmed to be extra-axial, solid, and mildly hemorrhagic. Resection was performed by initial internal debulking followed by circumferential microsurgical dissection of the lesion from the surrounding structures. After removal of the bulk of the tumor, a thin residual layer densely adherent to the patent right transverse sinus was intentionally left in place to avoid venous sinus injury. The tentorial attachment at the site of tumor involvement was partially resected. The postoperative course was uneventful, and the patient developed no new neurological deficits. Early postoperative MRI demonstrated near-total resection with a small residual remnant along the right transverse sinus, consistent with the intraoperative findings.

**Figure 2:**
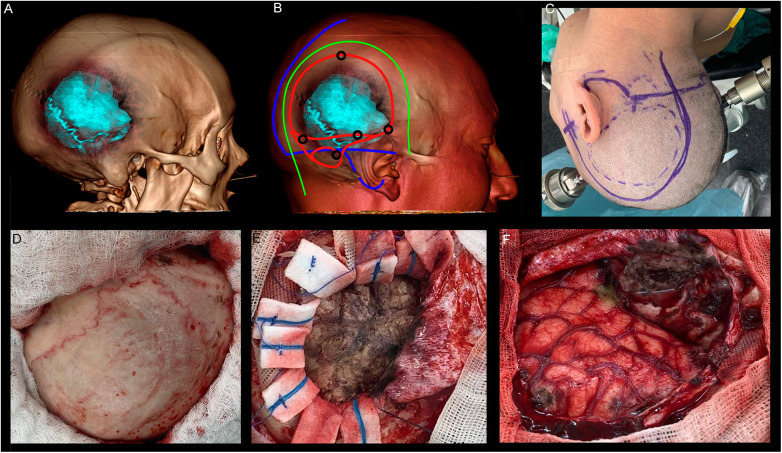
Preoperative planning and intraoperative findings. **(A,B)** Three-dimensional reconstructions illustrating tumor extent and key surgical landmarks. **(C)** Intraoperative positioning. **(D)** Intraoperative view after reflection of the skin flap, showing the osseous landmarks. **(E)** Intraoperative view of the extra-axial tumor before resection. **(F)** Operative field after tumor removal. The tentorial surface is exposed and was partially resected at the site of tumor attachment.

Histopathological examination showed a spindle-cell neoplasm with collagenized stroma and prominent branching staghorn-like vessels. Immunohistochemistry demonstrated diffuse CD34 positivity, EMA negativity, S100 negativity, focal progesterone receptor positivity, and a Ki-67 labeling index of <1%. Overall, the findings were consistent with SFT. STAT6 immunohistochemistry and NAB2::STAT6 fusion testing were not performed at the time of diagnosis (8). Given the intentional residual remnant along the transverse sinus and concern for long-term local control, the residual lesion was treated 6 weeks after surgery with single-fraction linear accelerator (LINAC)-based stereotactic radiosurgery after multidisciplinary discussion. Clinically, the patient improved progressively. Her headaches and other symptoms of intracranial hypertension resolved, and the visual deficit showed substantial recovery on serial Goldmann perimetry. At 6-month follow-up, MRI demonstrated reduction of the residual lesion, resolution of the midline shift, restoration of normal tonsillar morphology and position, and complete resolution of the C1 syrinx (Figure 3). On sagittal T2WI, the lowest cerebellar tonsil was located 1 mm above McRae's line, consistent with reversal of the preoperative caudal tonsillar descent. Postoperative spinal MRI, performed as part of the assessment for possible tumor dissemination, showed no additional syrinx segments, spinal lesion, arachnoiditis, tethering, or other spinal cause for the C1 cavity. Given the residual remnant and the known potential for late recurrence or dissemination of CNS SFT, continued long-term surveillance with serial contrast-enhanced brain MRI was planned, with repeat neuroaxis and extracranial imaging guided by clinical course and multidisciplinary follow-up.

**Figure 3:**
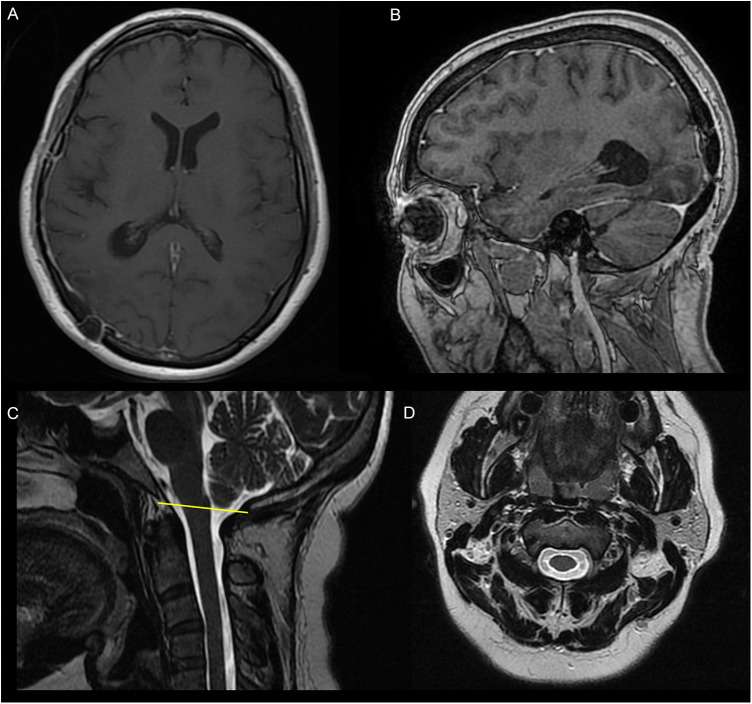
Six-month follow-up MRI after resection and adjuvant radiosurgery.**(A)** Axial post-contrast T1WI demonstrating resolution of the midline shift and mild ex vacuo dilatation of the right occipital horn and atrium, without hydrocephalus. **(B)** Right parasagittal contrast-enhanced T1WI through the site of dural attachment and dural resection, showing preservation of the transverse sinus and reduction of the residual tumor. **(C)** Midline sagittal T2WI of the craniocervical junction showing restoration of a rounded cerebellar tonsillar morphology and normal tonsillar position (McRae's line, yellow line), with complete resolution of the syrinx. **(D)** Axial T2WI at the C1 level confirming resolution of the syrinx.

## 3 Discussion

### 3.1 Observations

The main value of this case lies not in the tumor histology itself, but in the coherent radiological sequence it illustrates. A giant supratentorial posterior tentorial mass produced marked hemispheric mass effect, inferior deformation of the tentorial compartment, secondary caudal descent of the cerebellar tonsils, and a small upper cervical syrinx. After treatment of the causative lesion, the midline shift resolved, the tonsils returned to a normal position, and the syrinx disappeared. Taken together, this temporal and anatomical sequence argues against incidental coexistence and instead supports a reversible secondary craniocervical hydrodynamic disorder. Prior reports of acquired Chiari malformation with syringomyelia secondary to space-occupying lesions support the same interpretation, although most published cases have involved infratentorial rather than supratentorial pathology (2, 4–7).

### 3.2 Secondary Chiari-like state rather than primary Chiari malformation

Conceptually, this case is better interpreted as a secondary acquired Chiari-like state than as a primary Chiari I malformation. Although tonsillar ectopia is often described as an imaging diagnosis, the same radiographic pattern can arise through very different mechanisms, including congenital posterior fossa underdevelopment, cranial disproportion, CSF pressure disorders, and acquired disturbance of normal craniospinal CSF transmission (1, 3, 9). In this patient, several features point clearly to the latter explanation: symptom onset in adulthood, papilledema, a complete retrochiasmal visual field defect, a giant unilateral supratentorial lesion causing marked midline shift and tentorial deformation, no other structural abnormality of the posterior fossa, and postoperative reversal of both the tonsillar descent and the syrinx (2, 4–7). Alternative etiologies of tonsillar descent were also considered. There was no imaging evidence of congenital posterior fossa underdevelopment, craniovertebral junction malformation, basilar invagination, or other osseous abnormality suggesting a primary Chiari I malformation or bony crowding mechanism. Postoperative spinal MRI showed no tethering or other spinal abnormality that could suggest traction-related tonsillar descent. No clinical or imaging features suggested craniovertebral junction instability. In this context, the adult-onset, giant supratentorial mass with severe mass effect and postoperative reversal of both the tonsillar descent and the C1 cavity strongly favored a secondary acquired Chiari-like state. The preoperative peg-like configuration of the tonsils also supports this interpretation. On follow-up MRI, the tonsils had regained a rounded morphology. In Chiari-related states, peg-like tonsils are generally taken as a sign of crowding or impaction at the foramen magnum and have been associated with greater restriction of CSF flow than rounded tonsils (10, 11). In the present case, the change in tonsillar shape after treatment is therefore most consistent with reversal of secondary tonsillar impaction. This is not just a semantic distinction. If the hindbrain abnormality is secondary, then the foramen magnum finding is not the primary disease itself, but a downstream expression of a more proximal process. Earlier reports of supratentorial lesions causing an acquired Chiari-like state support the same view. Sheehan and Jane described resolution of tonsillar herniation and a cervical syrinx after resection of a parieto-occipital supratentorial tumor, without posterior fossa decompression (6). Similar reversibility has been reported with supratentorial AVMs, colloid cysts with hydrocephalus, and large meningiomas (4, 5, 7, 12). In this setting, posterior fossa decompression alone risks addressing a downstream manifestation rather than the actual driver of the process.

### 3.3 Intracranial compartmental pressure gradients and the craniocervical bottleneck

The mechanistic question is how a purely supratentorial lesion can generate a hindbrain-cervical pattern of tonsillar descent and syringomyelia. A simple downward-displacement model is likely insufficient. A more plausible explanation is that a giant supratentorial tentorial mass disturbs how pressure and pulsatile volume changes are transmitted across intracranial compartments and, in doing so, secondarily compromises the craniocervical junction. Under normal conditions, each systolic cardiac cycle transiently increases intracranial arterial volume. Because the cranial vault is effectively rigid, that increase must be buffered rapidly by displacement of venous blood and CSF, including caudal CSF movement through the foramen magnum into the spinal subarachnoid space (3, 9, 13–15). When a large lesion markedly distorts supratentorial geometry and deforms the tentorial compartment, that craniospinal compensatory exchange may become less efficient, particularly if the hindbrain is secondarily crowded at the foramen magnum (3, 13, 14). A related mechanism may also operate in focal lesions at the craniocervical junction, where local crowding and disturbance of CSF passage have been associated with cervical syringomyelia even in the absence of tonsillar descent or a primary spinal cord lesion (16, 17). In the present case, the problem is not merely one of reduced bulk CSF flow, but of altered compliance and abnormal pulse-pressure transmission across a narrowed interface. Oldfield et al. showed that in Chiari-associated syringomyelia, partial obstruction at the foramen magnum is accompanied by abnormal downward systolic movement of the tonsils, such that they behave as a piston and generate accentuated pressure waves in the cervical subarachnoid space (14). Heiss et al. later expanded this model by demonstrating that subarachnoid obstruction is associated with increased cervical pulse pressure and reduced compliance, thereby exposing the cord to repetitive exaggerated external pulsatile forces (13). More recent syntheses have emphasized that Chiari-related pathology cannot be understood from static morphology alone; altered CSF hydrodynamics and reduced craniospinal compliance are central to the process (3, 9).

Venous dynamics add another layer to this problem. A large hypervascular lesion closely related to the transverse sinus could have impaired venous outflow even without frank sinus thrombosis, thereby increasing venous pressure, reducing the efficiency of CSF absorption, and diminishing the reserve available for craniospinal pressure buffering (18). In that setting, venous congestion could have acted as a reinforcing component of the same compliance-hydrodynamic disorder. This interpretation is supported by reports in which posterior fossa venous congestion, cerebellar venous reflux, and distal dural sinus stenosis or thrombosis were linked to reversible tonsillar prolapse after restoration of venous drainage (19). It is also consistent with observations in syndromic craniosynostosis, where surgical expansion of the posterior cranial vault can increase dural sinus volume, improve venous drainage, and coincide with improvement of chronic tonsillar herniation and syringomyelia (20). More broadly, the association between aberrant venous collateral drainage, ventricular size, and intracranial pressure regulation supports a close interaction between venous outflow and intracranial fluid homeostasis (21).

Applied to the present case, the most coherent interpretation is that the giant supratentorial lesion altered intracranial compartmental mechanics sufficiently to convert the foramen magnum into a functional bottleneck. Once that occurred, rapid craniospinal pressure equalization would have become impaired, and the upper cervical subarachnoid space would have been subjected to abnormal transmitted pulse-pressure effects. This combined static-dynamic disturbance provides a plausible link between supratentorial mass effect, secondary tonsillar descent, and formation of a small upper cervical syrinx. It also explains why reversal of the upstream lesion, rather than posterior fossa decompression, was followed by normalization of tonsillar position and disappearance of the syrinx (Figure 4).

**Figure 4:**
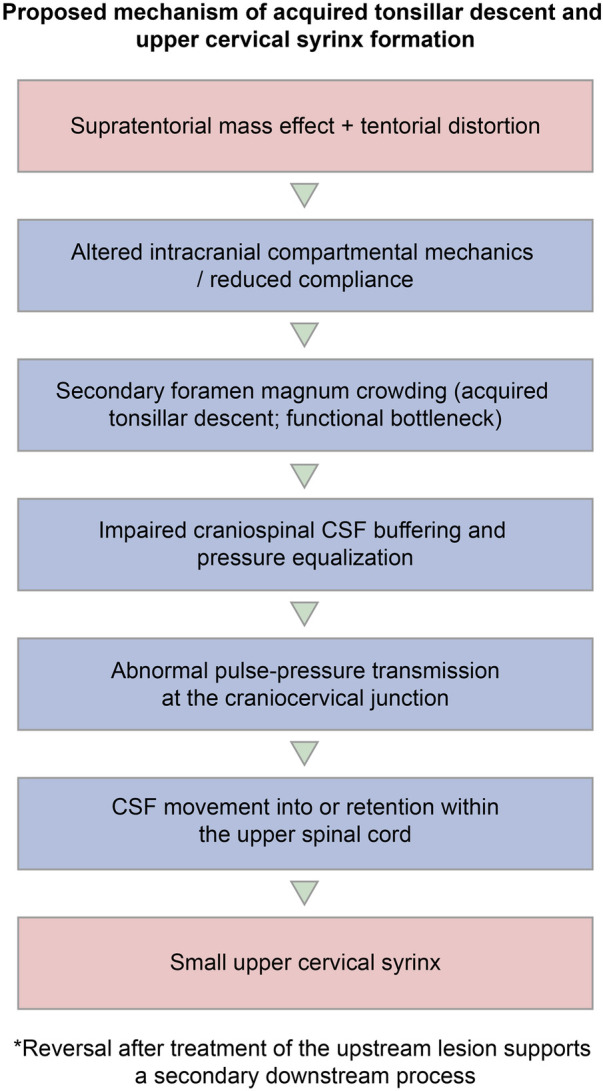
Proposed mechanism of acquired tonsillar descent and upper cervical syrinx formation in the present case. Supratentorial mass effect and tentorial distortion are hypothesized to alter intracranial compartmental mechanics and reduce compliance, leading to secondary foramen magnum crowding with acquired tonsillar descent and a functional craniocervical bottleneck. This may impair craniospinal CSF buffering and pressure equalization, promote abnormal pulse-pressure transmission at the craniocervical junction, and facilitate fluid movement into or retention within the upper cervical cord, resulting in a small upper cervical syrinx. Reversal after treatment of the upstream lesion supports a secondary downstream process.

### 3.4 Syringogenesis: historical theories and their modern relevance

Syringogenesis in Chiari-related disease remains incompletely resolved, and several classical and modern hypotheses deserve consideration. Gardner's hydrodynamic theory proposed that obstruction near the fourth ventricular outlets redirected pulsatile CSF into the central canal, the so-called “water-hammer” concept (22). Williams later emphasized a craniospinal pressure dissociation model, in which transient pressure gradients across the foramen magnum drove fluid into the cord and distended a communicating cavity (15). These models were foundational because they framed syringomyelia as a dynamic pressure disorder, but neither fully explains the contemporary adult pattern, in which a persistent fourth ventricle–syrinx communication is usually absent and syrinxes often regress after restoration of subarachnoid pathways rather than direct cavity drainage.

For the present case, however, a modern obstructive-pulsatile/compliance framework is more persuasive than a classic communicating-hydrosyringomyelia model. Oldfield and colleagues proposed that, in Chiari-related syringomyelia, partial blockage at the foramen magnum allows the tonsils to move downward with each systole and act as a piston, thereby generating accentuated pressure waves in the cervical subarachnoid space (14). Heiss and coworkers further argued that the resulting enlarged cervical subarachnoid pressure waves compress the cord externally, promote fluid entry into the spinal cord, and propagate syrinx fluid caudally with each heartbeat (13). Levine, however, criticized the assumption that CSF is simply driven into the cord by a tonsillar piston and emphasized the need to distinguish between the site of obstruction, the dynamics of extracellular fluid movement, and the structural properties of the cord itself (23). In other words, it may be less important to ask whether fluid comes from the fourth ventricle or from the subarachnoid space than to understand how abnormal pressure transmission changes the fluid balance within the spinal cord. Greitz took this further by proposing that altered flow-velocity and pressure relationships around the cord produce an imbalance favoring extracellular fluid accumulation, invoking Bernoulli/Venturi-like effects and intramedullary pulse-pressure differentials rather than simply filling a pre-existing central canal (24). Although aspects of Greitz's formulation remain debated, it redirects attention to the cord as a deformable tissue exposed to abnormal pulsatile loading, rather than just a passive receptacle.

Applied to this patient, no single historical model can be proven. There was no evidence of hydrocephalus or another finding suggesting a direct ventricular communication, whereas the lesion produced marked supratentorial distortion, secondary tonsillar descent, and a small upper cervical syrinx that resolved after treatment of the causative mass alone (3). Taken together, this pattern is most consistent with a reversible secondary syringomyelia driven by abnormal craniospinal pulse-pressure transmission and reduced compliance at a functionally narrowed foramen magnum. The most plausible sequence is therefore (Figure 4): supratentorial mass effect altered intracranial compartmental compliance and tentorial-hindbrain geometry; the foramen magnum became a functional bottleneck; craniospinal pressure equalization deteriorated; abnormal pulse-pressure transmission at the craniocervical junction promoted fluid movement into or retention within the upper cervical cord; and a small syrinx developed (9, 14, 23, 24).

### 3.5 Why the syrinx and tonsillar descent regressed

The reversal phase is as informative as the formation phase. If the syrinx were an autonomous intramedullary process, one would not expect the cavity to collapse or regress after treatment of a remote supratentorial lesion alone. Instead, the 6-month follow-up suggests that the cavity was maintained by an ongoing mechanical-hydrodynamic disturbance. Once the major supratentorial mass effect was relieved, the tentorial geometry, hindbrain configuration, and foramen magnum environment normalized sufficiently for the abnormal craniospinal pressure transmission to subside. In that setting, the force maintaining transmedullary or extracellular fluid accumulation would be expected to diminish, allowing gradual cavity collapse (2, 6, 13, 14).

Crucially, this correction was achieved without posterior fossa decompression. That observation reinforces the principle that in acquired cases, restoration of the upstream cranial mechanical environment may be sufficient to normalize the downstream hindbrain-cervical state. This is consistent with the systematic review by Wang et al. and prior supratentorial case reports in which treatment of the underlying lesion alone led to tonsillar ascent and syrinx regression (2).

### 3.6 Limitations

This report cannot distinguish definitively among competing models of syringogenesis. No cine phase-contrast MRI, direct intracranial or spinal pressure measurements, or quantitative compliance assessments were available. The relative contributions of global intracranial pressure elevation, local tentorial deformation, tonsillar piston effects, altered venous buffering, and transmedullary fluid movement therefore cannot be experimentally separated. The argument advanced here is inferential rather than demonstrative. In addition, the present report describes a single patient with a relatively small, high cervical syrinx and a specific pattern of supratentorial mass effect. The extent to which the same hydrodynamic sequence applies to larger syrinxes, to more chronic cases, or to lesions with different compartmental relationships remains uncertain. Nevertheless, the serial radiological evolution strongly supports a reversible secondary craniocervical hydrodynamic disorder and is difficult to explain satisfactorily by a coincidental or purely static model.

## 4 Conclusion

A giant supratentorial tentorial mass can generate a reversible acquired Chiari-like state with cervical syringomyelia through distortion of intracranial compartmental mechanics and secondary obstruction of craniocervical CSF transmission. In such cases, the syrinx is best understood as a downstream marker of abnormal craniospinal hydrodynamics rather than as a primary spinal lesion. Relief of the upstream lesion may be sufficient for normalization of tonsillar position and syrinx regression, without posterior fossa decompression.

## Data Availability

The original contributions presented in the study are included in the article, further inquiries can be directed to the corresponding author.
